# ﻿Phylogenetic analysis of the genus *Semioscopis* (Lepidoptera, Depressariidae), with description of a new species from China

**DOI:** 10.3897/zookeys.1265.176105

**Published:** 2025-12-30

**Authors:** Jia-Xin Wang, Xiao-Ju Zhu, Yun-Li Xiao

**Affiliations:** 1 School of Forestry, Northeast Forestry University, Harbin 150040, China Northeast Forestry University Harbin China; 2 Hubei Key Laboratory of Economic Forest Germplasm lmprovement and Resources Comprehensive Utilization, Huanggang Normal University, Huanggang, Hubei, 438000, China Huanggang Normal University Huanggang China; 3 College of Plant Protection, Shandong Agricultural University, Tai’an, Shandong 271018, China Shandong Agricultural University Tai’an China

**Keywords:** Dabie Mountains, DNA barcode, Hubei, moths, new species, taxonomy

## Abstract

This study describes *Semioscopis sinicella* Wang, Zhu & Xiao, **sp. nov.** of the genus *Semioscopis* Hübner, 1825 (Lepidoptera, Depressariidae) from China. The new species is similar in external morphology and male genitalia to the European *S. avellanella* (Hübner, 1793) and the Japanese *S. similis* Saito, 1989, but it can be distinguished in female genitalia mainly by the distinctly shorter sclerotised section of the ductus bursae and the length ratios of the ductus bursae and corpus bursae to the papillae anales. Morphological descriptions and illustrations of the new species are provided. Furthermore, a phylogenetic analysis based on COI gene sequences using IQ-tree supports *S. sinicella* sp. nov. as a monophyletic lineage and further divides the genus *Semioscopis* into seven species groups.

## ﻿Introduction

The genus *Semioscopis* Hübner, 1825 (Lepidoptera, Depressariidae) was established with *Tortrix steinkellneriana* Denis & Schiffermüller, 1775 as its type species. It currently comprises 13 valid species worldwide, with seven distributed in the Palearctic region (Denis and Schiffermüller 1775; Hübner 1793; Thunberg 1794; Saito 1989; Lepiforum 2025; Ponomarenko and Koshkin 2025) and six in the Nearctic region (Clemens 1863; Walsingham 1882; Dyar 1902; Clarke 1941; Hodges 1974). Early records from other regions have been subject to taxonomic revision: Semioscopis (Eulechria) lividella Meyrick, 1883 from Australia was subsequently synonymised with *Scotodryas lividella*, and Semioscopis (Epigraphia) osthelderi (Rebel, 1936) from Turkey was transferred to the genus *Luquetia* (Osthelder 1936; Buchner and Corley 2025). The first confirmed Asian representatives were reported by Saito (1989), who documented the genus in Japan and described two species, *S. similis* Saito, 1989 and *S. japonicella* Saito, 1989. More recently, Koo et al. (2023) recorded the genus in South Korea based on a specimen initially identified as *S.* “*japonicella*”. Subsequent detailed morphological examination by Ponomarenko and Koshkin (2025) first revealed that specimens previously identified as *S.* “*japonicella*” from the Russian Far East actually represent a distinct species, described as *S. fareastenica* Ponomarenko & Koshkin, 2025. This distinction was initially based on genital morphology, as genetic data for the true *S. japonicella* from Japan were unavailable (see Ponomarenko and Koshkin 2025). Their molecular analysis of the COI gene later confirmed that the specimen from South Korea also belongs to *S. fareastenica*. Their study also reported the first record of *S. strigulana* (Denis & Schiffermüller, 1775) in Primorskii Krai and provided a comprehensive checklist of *Semioscopis* species in East Asia.

In this study, we present the first confirmed record of *Semioscopis* from China and describe *Semioscopis sinicella* sp. nov. collected from the Dabie Mountains in Hubei Province, China. The description is supported by integrated morphological evidence and COI DNA barcode data. Furthermore, based on a phylogenetic analysis and review of previous taxonomic works, we discuss and refine the species group classification within the genus.

## ﻿Material and methods

### ﻿Specimen collection and morphological examination

Specimens of the new species described in this study were collected via light trapping using a 450 W high-pressure mercury lamp in the Dabie Mountains, Hubei Province, China.

The holotype and paratypes of *Semioscopis sinicella* sp. nov. are deposited in the Shandong Agricultural University (**SDAU**). Genitalia were dissected and slide mounted following the protocol of Li (2002). Morphological terminology used in the descriptions primarily follows Saito (1989) and Mou (2012). Photographs of living adults of *Semioscopis* species from around the globe, used in this study, were obtained and utilised with appropriate authorisation (Fig. 2). Images of adults and genitalia were captured using a Canon EOS 70D camera equipped with an EF 180mm F/3.5L USM lens and a Nexcope NE930 microscope, respectively. All images were subsequently refined using Adobe® Photoshop® 2023 software. Type locality map created using QGIS Desktop 3.40.0.

### ﻿DNA extraction and data acquisition

**Figure 1.**
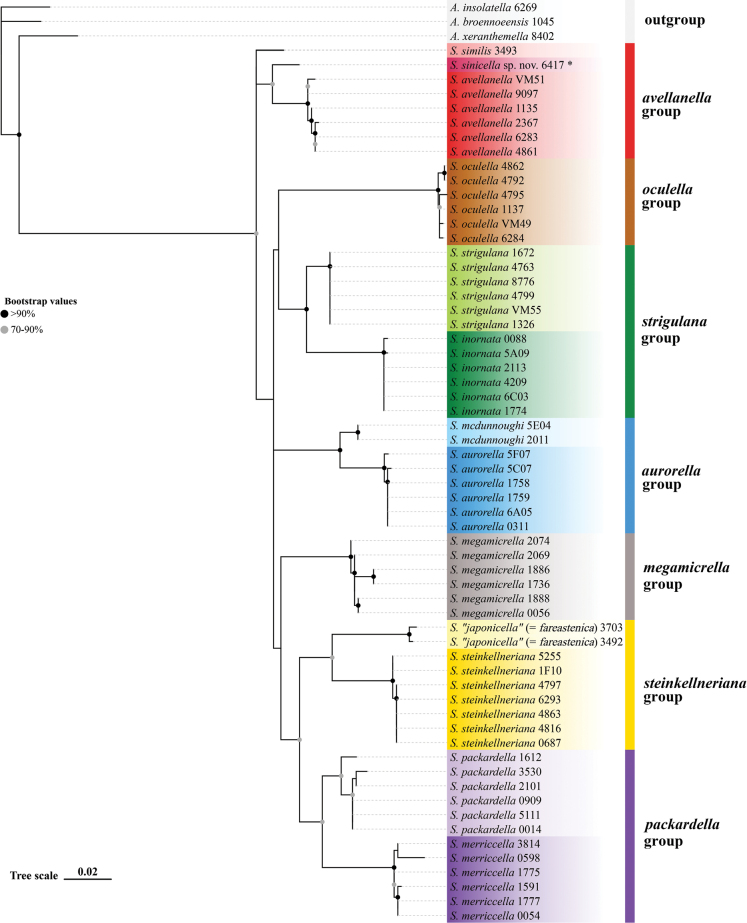
Maximum-likelihood IQ-Tree phylogenetic tree of 13 *Semioscopis* species based on the DNA barcode sequences (COI) and rooted with the genus *Agonopterix* as the outgroup.

DNA was extracted from the legs of freshly obtained specimens of the new species using the TIANamp Genomic DNA Kit (DP304). The 658 bp barcode region of the cytochrome c oxidase subunit I (COI) gene was amplified using the primers LepF1 and LepR1 (Hajibabaei et al. 2006). The PCR products were sent to Tianyi Huayu Gene Co., Ltd (Wuhan, China) for bidirectional sequencing with the same primers. Phylogenetic reconstruction was based on a comprehensive global dataset of 63 COI sequences, encompassing 13 recognised species of *Semioscopis*, except for *Semioscopis japonicella* Saito, 1989. Additionally, three species from the genus *Agonopterix* Hübner, 1825 were included as the outgroup (Fig. 1). The four-digit number following each species name corresponds to the last four digits of the BOLD sample ID or GenBank accession number. Values at the nodes indicate bootstrap values. All sequences are publicly accessible via BOLD Systems (https://boldsystems.org/) or GenBank (https://www.ncbi.nlm.nih.gov/) (Table 1).

**Table 1.** Sample information for *Semioscopis* species and outgroup.

Species	Location	NCBI and BOLD accession no.
*Semioscopis similis* Saito, 1989	Russia	TLMF Lep 23493
*Semioscopis sinicella* **sp. nov.**	China, Hubei	OR596417
*Semioscopis avellanella* (Hübner, 1793)	Austria	TLMF Lep 16283
	Switzerland	TLMF Lep 04861
	Germany	BC ZSM Lep 29097
	Finland	MM01135
	Norway	NorBOL LepVM51
	Italy	TLMF Lep 02367
*Semioscopis oculella* (Thunberg, 1794)	Austria	TLMF Lep 16284
	France	TLMF Lep 04792
	Switzerland	TLMF Lep 04795
	France	TLMF Lep 04795
	Finland	MM01137
	Norway	NorBOL LepVM49
*Semioscopis strigulana* (Denis & Schiffermüller, 1775)	Switzerland	TLMF Lep 04799
	Germany	BC ZSM Lep 61326
	Austria	TLMF Lep 14763
	Italy	TLMF Lep 11672
	Finland	MM08776
	Norway	NorBOL LepVM55
*Semioscopis inornata* Walsingham, 1882	United States	BIOUG01046-C03
	United States	CCDB-29465-A09
	Canada	CGWC-4209
	Canada	jflandry0088
	Canada	jflandry1774
	Canada	MDH002113
*Semioscopis mcdunnoughi* Clarke, 1941	United States	122096261Apr2011
	Canada	CCDB-22975-E04
*Semioscopis aurorella* Dyar, 1902	United States	BIOUG01046-A05
	Canada	jflandry1758
	Canada	jflandry1759
	Canada	BIOUG33515-C07
	Canada	HLC-10311
	Canada	BIOUG35065-F07
*Semioscopis megamicrella* Dyar, 1902	Canada	jflandry0056
	Canada	jflandry1736
	Canada	jflandry1886
	Canada	jflandry1888
	Canada	MDH002074
	Canada	MDH002069
*Semioscopis steinkellneriana* (Denis & Schiffermüller, 1775)	Austria	TLMF Lep 16293
	United States	UKLB41F10
	Switzerland	TLMF Lep 04797
	Austria	TLMF Lep 14816
	Norway	NHMO-DAR-5255
	Germany	BC ZSM Lep 50687
*Semioscopis* “*japonicella*” (= *fareastenica*)	Russia	TLMF Lep 23492
	South Korea	OQ573703
*Semioscopis packardella* Clemens, 1863	Canada	BL0014
	United States	DNA-ATBI-3530
	United States	FRC41612
	Canada	jflandry2101
	Canada	CNCLEP00025111
	Canada	MDH000909
*Semioscopis merriccella* Dyar, 1902	United States	FRC00598
	United States	FRC01591
	Canada	CGWC-3814
	Canada	jflandry0054
	Canada	jflandry1760
	Canada	jflandry1775
*Agonopterix insolatella* Buchner, 2021	Algeria	MT406269
*Agonopterix broennoeensis* (Strand, 1919)	Finland	MZ611045
*Agonopterix xeranthemella* Buchner, 2018	Turkey	MK308402

**Figure 2.**
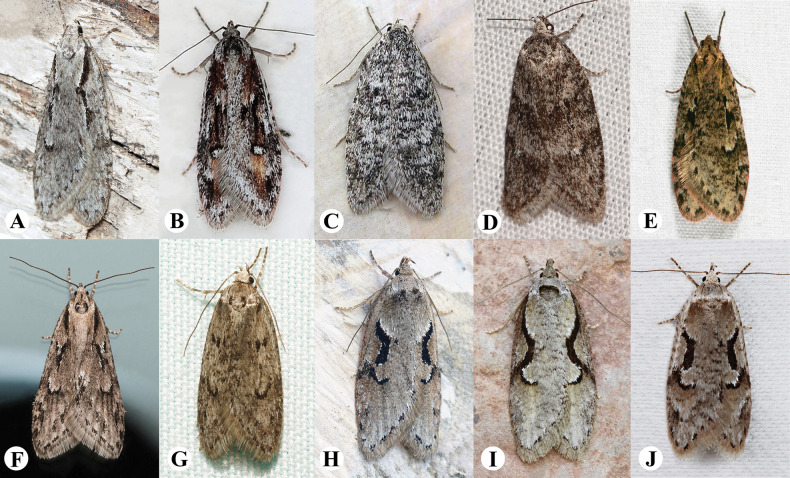
Photographs of living adults of *Semioscopis* species from around the globe. **A.***S. avellanella*, Moscow, Russia (photo: Ilya G. Ustyantsev); **B.***S. oculella*, Moscow, Russia (photo: Andrey Ponomarev); **C.***S. strigulana*, Moscow, Russia (photo: Andrey Ponomarev); **D.***S. inornata*, Quebec, Canada (photo: Claudette Cormier); **E.***S. mcdunnoughi*, British Columbia, Canada (photo: John Reynolds); **F.***S. aurorella*, United States (photo: Brighton Lee); **G.***S. megamicrella*, United States (photo: Kyle Klotz); **H.***S. steinkellneriana*, Moscow, Russia (photo: Ilya G. Ustyantsev); **I.***S. packardella*, Ontario, Canada (photo: Michael H. King); **J.***S. merriccella*, Ontario, Canada (photo: Michael H. King).

### ﻿Phylogenetic analysis and genetic divergence

Phylogenetic analysis was conducted using the PhyloSuite v. 1.2.3 (Zhang et al. 2020; Xiang et al. 2023). Specifically, a maximum-likelihood (ML) tree was constructed with IQ-TREE v. 2.2.0 (Nguyen et al. 2015), which was integrated into the software. The optimal nucleotide substitution model was automatically selected by the Auto function in IQ-TREE. Nodal support was assessed with 5000 ultrafast bootstrap replicates (Minh et al. 2013). The final phylogenetic tree was visualised and refined using the online tool Chiplot (https://www.chiplot.online/) (Xie et al. 2023). The COI sequences were translated into amino acid sequences, aligned using MUSCLE v. 3.8 (Edgar 2004), and manually corrected. Intraspecific and interspecific genetic distances were calculated based on the Kimura 2-parameter (K2P) model (Kimura 1980) using MEGA v. 11.0 (Tamura et al. 2021). “*N*” represents the number of sequences for each species in Table 2.

## ﻿Results

### ﻿DNA sequence analysis

Combined with sequences from the outgroup taxa, a final dataset of 63 sequences from 13 *Semioscopis* species was analyzed. The maximum-likelihood phylogeny (Fig. 1) showed that the new species, *Semioscopis sinicella* sp. nov., formed a well-supported monophyletic lineage. It clustered together with *S. similis* from Russia and *S. avellanella* from Europe, yet exhibited significant genetic divergence from them, with average distances of 2.17% and 3.04%, respectively. For comparison, the average genetic distance between the recognised sister species *S. mcdunnoughi* and *S. aurorella* was 2.35% (Table 2).

**Table 2.** Average Kimura 2-parameter genetic distance in percentage, calculated within (in bold) and among *Semioscopis* species.

	1	2	3	4	5	6	7	8	9	10	11	12	13
1. *S. similis* (*N* = 1)	—												
2. *S. sinicella* sp. nov. (*N* = 1)	2.17	—											
3. *S. avellanella* (*N* = 6)	3.04	2.59	**0.33**										
4. *S. oculella* (*N* = 6)	5.96	6.41	6.16	**0.37**									
5. *S. strigulana* (*N* = 6)	3.27	4.08	3.78	5.63	**0.00**								
6. *S. inornata* (*N* = 6)	4.46	5.12	5.59	7.12	3.33	**0.10**							
7. *S. mcdunnoughi* (*N* = 2)	3.92	4.74	4.93	7.32	3.75	5.26	**0.00**						
8. *S. aurorella* (*N* = 6)	5.21	5.76	5.84	8.49	5.25	6.74	2.35	**0.15**					
9. *S. megamicrella* (*N* = 6)	4.66	4.82	4.31	7.06	4.59	5.73	5.00	5.30	**0.54**				
10. *S.* “*japonicella*” (= *fareastenica*) (*N* = 2)	5.13	5.54	6.18	8.37	6.11	7.16	6.66	7.87	6.67	**0.46**			
11. *S. steinkellneriana* (*N* = 7)	4.51	5.00	5.93	7.61	5.49	5.97	5.85	6.71	5.98	4.78	**0.07**		
12. *S. packardella* (*N* = 6)	4.13	4.76	5.01	7.31	4.56	5.93	5.12	6.14	4.66	5.03	5.35	**0.55**	
13. *S. merriccella* (*N* = 6)	4.59	5.64	5.84	7.02	5.97	7.10	6.37	7.80	6.15	5.40	5.71	3.90	**0.58**

### ﻿Taxonomic account

#### ﻿ Semioscopis

Taxon classificationAnimaliaLepidopteraDepressariidae

Hübner, 1825

1741A5B9-71C2-5A40-AD29-43A4CF803EDC

Semioscopis Hübner, 1825: 402. Type species: Tortrix steinkellneriana Denis & Schiffermüller, 1775.
Epigraphia Stephens, 1829: 49. Type species: Tortrix steinkellneriana Denis & Schiffermüller, 1775.

##### Distribution.

China, South Korea, Russia, Japan, Turkey, Algeria, Australia, Europe, and North America.

#### ﻿ Semioscopis sinicella

Taxon classificationAnimaliaLepidopteraDepressariidae

Wang, Zhu & Xiao sp. nov.

7DF958DE-F262-56E3-ABC8-B2176734BB0B

https://zoobank.org/4D14F763-D390-483F-A924-4684C6CDC6BB

[中国符宽蛾] Figs 3–8, 9

##### Material examined.

***Holotype*.** China • ♂; Hubei province, Wujiashan, Dabie Mountain National Nature Reserve, Yingshan County, 31.1032°N, 115.7726°E, alt. 1549 m, 2.IV.2023, Jia-Xin Wang & Peng Yu leg., genitalia slide no. Lep2572♂. ***Paratype*.** China • 1♀, same data as holotype, Jia-Xin Wang & Peng Yu leg., genitalia slide no. Lep2573♀.

##### Diagnosis.

This species belongs to the Palearctic *avellanella* group along with two other species: *Semioscopis avellanella* (Hübner, 1793) from Europe and *S. similis* Saito, 1989 from Japan. The species is similar to *S. avellanella* and *S. similis* in appearance and male genitalia, but it can be distinguished in female genitalia mainly by having the basal 1/3 of the ductus bursae sclerotised, length of the ductus bursae about 1.2 times the papillae anales, and length of the corpus bursae about 1.6 times the papillae anales. In *S. avellanella* the basal 2/3 of the ductus bursae is sclerotised, and the ductus bursae and corpus bursae length are about 1.5 times and 1.9 times of the papillae anales, respectively. In *S. similis*, the distal 2/3 of the ductus bursae is sclerotised, and the ductus bursae and corpus bursae lengths are about 0.7 times and 1.2 times the papillae anales, respectively.

##### Description.

**Figures 3–8.**
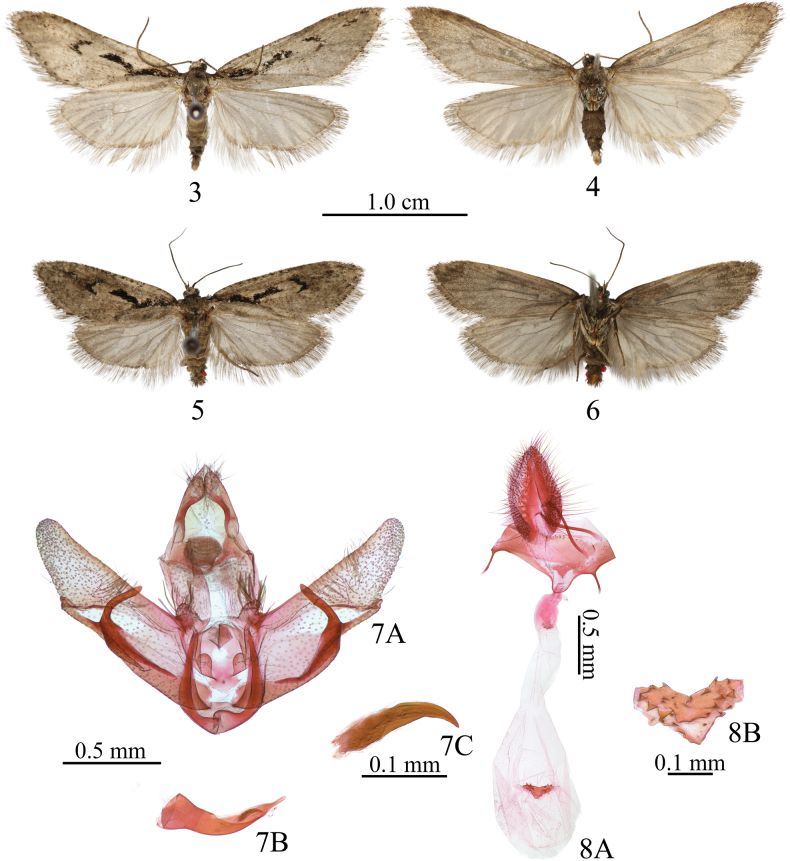
*Semioscopis sinicella* sp. nov. **3, 4.** Adult of male, holotype; **5, 6.** Adult of female, paratype; **7.** Male genitalia, holotype; **7A.** Male genitalia without aedeagus; **7B.** Aedeagus; **7C.** Cornutus; **8.** Female genitalia, paratype; **8A.** Female genitalia; **8B.** Signum. Scale bars: 1.0 cm (**3–6**); 0.5 mm (**7A, B, 8A**); 0.1 mm (**7C, 8B**).

**Adult** (Figs 3–6). Wingspan female 22 mm, male 26 mm. Head grey to dark grey, scales with greyish-white tips on vertex; face fuscous. Antenna pale fuscous. Labial palpus pale brownish grey; basal segment with slightly pale ochreous scales; second and third segments suffused with pale brownish-grey scales on outer margin, third segment about 1/3 length of second segment. Thorax brownish grey to fuscous, covered with greyish-white scales. Forewing grey, covered with white scales and dotted with black scales, especially on costal margin and termen; a longitudinal black streak in cell, slightly obliquely extending from base of costa to before middle of cell, then turned upwards in a obtuse angle, its end reaching to distal 3/4 of cell; a black crescentric blotch at end of cell; terminal dots black; fringe grey, with somewhat darker basal and subapical shades. Hindwing pale grey, with a darker basal shade. Legs dark brown; femora and tibiae mixed with pale-ochreous and greyish-white scales; tarsi of mid- and hindlegs mixed with pale-ochreous scales, tarsus of hindleg covered with long grey hairs dorsally, interspersed with yellowish-white scales on basal tarsomere.

***Male genitalia*** (Fig. 7A–C). Uncus almost equal in length to tegumen, gradually narrowed from base to middle, then gradually widened from middle to sub-rounded apex, socii small, broad, and setose. Gnathos rounded, densely covered with tiny spines; lateral arm triangular in basal 2/5 and slender in distal 3/5. Valva subrectuangular in basal 2/3, gradually narrowed in distal 1/3, apex rounded. Transtilla banded; transtilla lobes subrhombic, covered with long hairs. Costa nearly straight. Sacculus heavily sclerotised, slightly wider at base, extending to distal 1/3 to form long process bent at right angle and short process extending parallel; short process 4/5 length of long process. Juxta with a horseshoe-shaped cut, medial on posterior margin; lateral margins with V-shaped incision at anterior 1/4; lateral lobes semicircular, arising from anterior half, setose marginally. Vinculum broadly banded, with a slight posterior projection. Aedeagus gradually tapering from wide base to apex, curved medially approximately at 130°; cornutus composed of a long, stout, curved spine and additional small and medium-sized appressed spines attached near its base.

**Figures 9–13.**
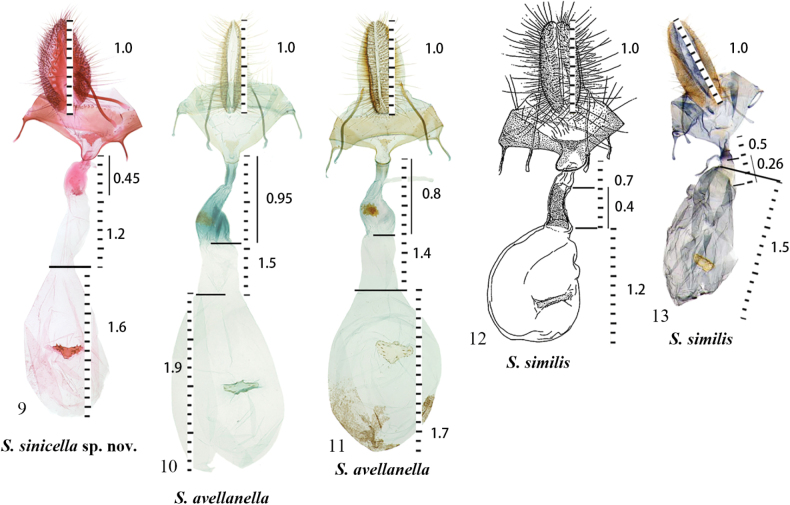
Comparative diagram illustrating the length ratios of the ductus bursae and corpus bursae in female genitalia of the *avellanella* species group. **9.***S. sinicella* sp. nov., paratype, China; **10, 11.***S. avellanella*, Austria (supplied by Peter Buchner); **12.***S. similis*, paratype, Japan (after Saito 1989); **13.***S. similis*, Japan (after Ponomarenko and Koshkin 2025). All lengths are standardised relative to the length of the papillae anales (shown as the scale bar).

***Female genitalia*** (Figs 8A, B, 9). Apophyses anteriores about 1/3 length of apophyses posteriores. 8^th^ sternite U-shaped, produced anteriorly. Lamella antevaginalis triangular. Antrum enlarged, oval. Ductus bursae thick, short, with basal 1/3 sclerotised; length about 1.3 times of papillae anales. Corpus bursae nearly oval, length about 1.7 times of papillae anales; signum situated at about half of corpus bursae, transversely dentate, and triangularly produced medially.

##### Distribution (Fig. 14).

China (Hubei).

**Figure 14.**
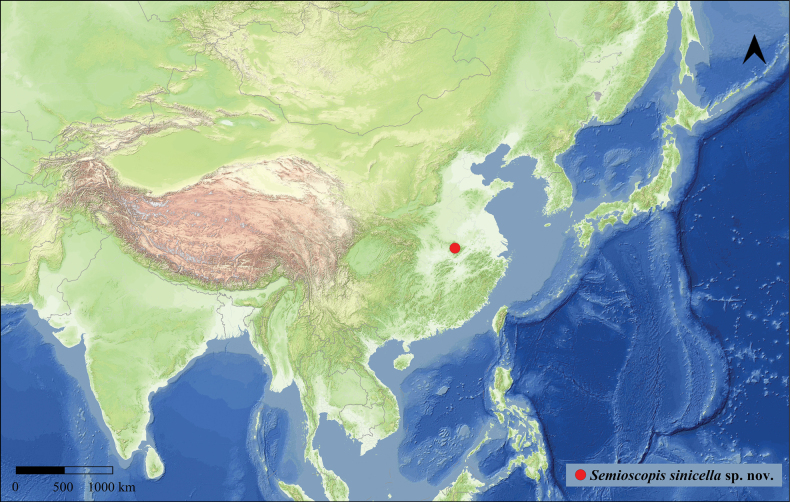
Type locality map of *Semioscopis sinicella* sp. nov. in China.

##### Etymology.

The specific name *sinicella* is derived from Latin word *sinicum* (= China) and –*cella* (a common feminine noun suffix), in reference to China.

##### Remarks.

The male is slightly larger than the female (holotype: 26.0 mm; paratype: 22.0 mm), with a more acute forewing apex and overall paler colouration. Because the description of *S. sinicella* sp. nov. is based on a single pair of specimens (holotype male and paratype female), the range of intraspecific variability in both morphological and molecular characters remains unknown. Future collections of additional material will be essential to assess this variability and to further confirm the stability of the diagnostic features presented here.

## ﻿Discussion

Integrated evidence from both morphology and molecular data robustly supports the establishment of *Semioscopis sinicella* sp. nov. as a distinct species. The new species is morphologically similar to the European *S. avellanella* and the Japanese *S. similis* in external appearance and male genitalia (Figs 9–13). However, it can be reliably distinguished by characters of the female genitalia, specifically the position of the sclerotised ductus seminalis and the relative ratios of the lengths of the ductus seminalis and corpus bursae to the papillae anales. Molecular data further corroborate its specific status. In the maximum-likelihood phylogeny, *S. sinicella* sp. nov. forms a well-supported monophyletic lineage (Fig. 1). The minimum genetic distances between the new species and *S. similis* (2.17%) and between the new species and *S. avellanella* (3.04%) are significant and comparable to those observed between other recognised sister species within the genus (Table 2).

Combined with previous morphological studies, our molecular results allow a revised classification of *Semioscopis* into seven species groups (Fig. 1). The *avellanella* group includes *S. sinicella* sp. nov., *S. similis* Saito, 1989, and *S. avellanella* (Hübner, 1793), which share high morphological similarity and exhibit low interspecific genetic divergence (2.17–3.04%). This grouping aligns with the concept of Saito (1989). The *oculella* group is represented solely by *S. oculella* (Thunberg, 1794), a morphologically distinct species that occupies an isolated phylogenetic position. The *steinkellneriana* group comprises *S. steinkellneriana* (Denis & Schiffermüller, 1775), *S. japonicella* Saito, 1989, and *S. fareastenica* Ponomarenko & Koshkin, 2025, a delineation supported by both molecular phylogeny (however, no data for *S. japonicella* available) and morphological characters (Saito 1989; Lepiforum 2025; Ponomarenko and Koshkin 2025). Four additional Nearctic groups previously proposed by Hodges (1974) are corroborated here: the *strigulana* group (*S. strigulana* (Denis & Schiffermüller, 1775) and *S. inornata* Walsingham, 1882), the *aurorella* group (*S. aurorella* Dyar, 1902 and *S. mcdunnoughi* Clarke, 1941), and the *packardella* group (*S. packardella* Clemens, 1863 and *S. merriccella* Dyar, 1902). The *megamicrella* group is retained as monotypic, containing only *S. megamicrella* Dyar, 1902.

The revised classification proposed herein, buttressed by the concurrent discovery of *S. sinicella* sp. nov. in central China, provides a synthesised morphological and molecular perspective. This finding makes it imperative to conduct further extensive systematic surveys across Asia to ultimately elucidate the true diversity and complex biogeography of the genus *Semioscopis*.

## Supplementary Material

XML Treatment for Semioscopis

XML Treatment for Semioscopis sinicella

## ﻿Additional information

### Use of AI

No use of AI was reported.

### Author ORCIDs

Jia-Xin Wang https://orcid.org/0009-0009-0412-1313

Xiao-Ju Zhu https://orcid.org/0000-0002-4590-8480

Yun-Li Xiao https://orcid.org/0000-0002-6782-4229

### Data availability

All of the data that support the findings of this study are available in the main text.
